# Annotations

**Published:** 1887-04-09

**Authors:** 


### Annotations.

The National Pension Fund.

Our proposal to establish a National Pension Fund for hospital officials and nurses, is growing in public favour, and will prove an undoubted success, providing those chiefly interested are true to themselves. We are rejoiced to see that *The Lancet* and *British Medical Journal* have both extended their support to this movement; the latter journal having advocated the establishment of such a Fund in an able article published last October. The National Pension Fund, which we are now aiding to render a success, was founded some years ago by Mr. Burdett, who had the scheme submitted to an independent actuarial authority for criticism before forwarding it to the various hospital authorities throughout the country. Subsequently every hospital matron and the heads of all the Nursing Associations were invited to co-operate, and some two thousand five hundred matrons and nurses signified their willingness to join, but the latter intimated they could not pay more than £2 per annum to a Pension and Sick Fund. Five pounds a year was found to be about the average payment necessary to secure £25 a year pension at fifty to fifty-five years of age. This difficulty was not unexpected, and negotiations were opened and have since proceeded with various hospital committees, which have resulted in the discovery that if every hospital will pay its head-nurses at least £25 per annum and find them in uniform and washing, the £5 for the Pension Fund can be spared. We shall publish the outline of the National Pension Fund scheme in due course, but meanwhile we invite all who are interested to state their views and wishes on the subject. Names are coming in fast to Mr. Collins, the Secretary of the Hospitals Association, Norfolk House, W.C., to whom lists should be sent without delay. Lady Superintendents and Matrons are bringing the question under the notice of their nurses, and several have sent a long list usually comprising the names of the majority, and in some cases of all, the members of the staff employed in particular institutions. Other classes of hospital officials do not seem to be interested in the matter of securing a pension for themselves, if we may judge by the fact that they have so far almost entirely held aloof from the movement. The Secretaries of St. Mary's and University College Hospitals, Mr. Michelli and Mr. Nixon, have set an example in coming forward to advocate the establishment of this fund, which will no doubt be largely followed by metropolitan and provincial secretaries, as soon as they understand the proposal, and become interested in the fund. To all who desire the establishment of a National Fund, we have but one more word to say at present, "Register, register, register." If four or five thousand names are sent in, then a great meeting can be called, and a National Pension Fund will soon be placed upon a sound financial and lasting basis.

Private Wards for Lying-in Cases.

It is not generally known that arrangements have recently been made at the British Lying-in Hospital, Endell Street, Bloomsbury, to admit paying patients to private wards in a separate part of the buildings. Each patient has a private room to herself, comfortably furnished. The payment is two guineas per week, including all medical attendance, nursing, and board. It is pointed out by the friends of the British Lying-in Hospital that there are many members of the professional classes, and those whose avocations prevent them from forming permanent homes and who live mostly in lodgings, who would be glad to secure the very best accommodation and attention at what must always be a time of anxiety to the mother of a family and her friends. It is urged that the inhabitants of small houses, already crowded with a young family, might also feel glad to relieve the mother from the inconveniences and anxieties attendant on a confinement at home. The mischief sometimes arising to her who has to return to the headship of a large household before she is strong enough to undertake the work, is dwelt upon, and it is urged that recovery would be more rapid and complete in the hospital, with its quiet, restful surroundings, than amidst the bustle and turmoil of the confined home. We admit that there is some force in the points urged in favour of private wards for lying-in cases. At the same time there are social, hygienic, and general reasons why such a system should not be generally encouraged, and which would negative the use of a private ward by the majority of English mothers. No doubt a private hospital ward is better than the ordinary lodging, and under certain circumstances it will wisely be preferred, as the least of two evils. The English woman's home we believe, however, will be regarded by herself as well as by her friends, as the right and best place for her in times of sickness, as well as in times of health.

Locum Tenens.

Medical men, thinking of much-needed holidays, are often deterred by the difficulty of finding suitable temporary substitutes. Residents in hospitals, and especially in small hospitals, find equal difficulty for the same reason. On the other hand, there are many young doctors of excellent character who would only be too glad to see a little family and hospital practice. The question is, how to bring the two together. According to a suggestion, we propose to give the names of those requiring *locum tenens* on the one hand, and of qualified men of character and ability on the other, in our "Notes and News" column from week to week. Any inquiries on the subject will be answered in future numbers.

